# Sequential Gating of Ryanodine Receptors Underlies the Development of Calcium Sparks in Frog Skeletal Muscle

**DOI:** 10.3390/biom16060910

**Published:** 2026-06-19

**Authors:** Henrietta Cserne Szappanos, László Zsolt Szabó, Ildikó Balatoni, Martin F. Schneider, László Csernoch, Péter Szentesi

**Affiliations:** 1 Department of Biochemistry and Molecular Biology, School of Medicine, University of Maryland, Baltimore, MD 20742, USA; henrietta.csernedrszappanos@qimrb.edu.au (H.C.S.);; 2 Department of Physiology, Medical Faculty, University of Debrecen, 4032 Debrecen, Hungary; csl@edu.unideb.hu; 3 Department of Electrical Engineering, Sapientia Hungarian University of Transylvania, 540485 Târgu Mureş, Romania; lszabo@ms.sapientia.ro; 4 Clinical Center, University of Debrecen,4032 Debrecen, Hungary; balatoni@med.unideb.hu; 5 HUN-REN Cell Physiology Research Group, University of Debrecen, 4032 Debrecen, Hungary

**Keywords:** skeletal muscle, frog, excitation–contraction coupling, ryanodine receptor, calcium spark, membrane depolarization, caffeine

## Abstract

Calcium sparks can arise as both voltage-dependent and voltage-independent ligand-activated release events in amphibian skeletal muscle. To assess their gating behavior, calcium sparks were recorded from intact frog skeletal muscle fibers using high-temporal-resolution confocal microscopy (line scans: 15 and 50 µs/line). Sparks were triggered by 1 mmol/L caffeine to open ryanodine receptors (RyRs) or by subthreshold depolarization to a −65 mV membrane potential to activate dihydropyridine receptors (DHPRs). Both treatments increased the frequency of sparks and altered their morphology. The sparks were significantly greater after caffeine treatment than in depolarized cells. The signal mass of sparks (i.e., the amount of calcium released) resembled the amplitude in shape. Additionally, the calcium release flux followed a staggered function during the activation of sparks. The detailed analysis of the sparks’ time profile revealed that the events were activated in a stepwise manner. The average step size (in F/F_0_; 0.071 ± 0.003) remained constant regardless of the scanning speed. The number of steps during the activation of sparks followed a linear function based on the spark’s amplitude. Our results suggest that the activation of neighboring release units may occur sequentially, and the amplitude of the sparks depends linearly on the number of activated RyR channels.

## 1. Introduction

Skeletal muscle fibers use changes in intracellular calcium levels to regulate the interaction among contractile proteins, ultimately producing force. In vertebrates, calcium ions (Ca^2+^) are released from their storage site, the sarcoplasmic reticulum (SR), via calcium channels, such as the ryanodine receptor (RyR), following depolarization of the surface and transverse (T)-tubular membrane [1]. The voltage sensors in the T-tubule membrane, the dihydropyridine receptor (DHPR), and the RyR are arranged in clustered, highly organized formations [2]. This structure in amphibians features a double row of RyR in the SR membrane and a similar arrangement of tetrads of DHPR in the T-tubular membrane but with only every second RyR in each row having an opposing DHPR tetrad [2].

It is now widely accepted that the depolarization-driven conformational change in the DHPR causes the opening of the adjacent physically interacting RyR [3], while RyRs that lack direct connections to tetrads can be opened either through interaction with their neighboring RyR (coupled gating; [4]) or by calcium ions released from these neighboring channels (calcium-induced calcium release; CICR; [5]). However, in addition to the RyRs found in the junctional SR (jSR), parajunctional RyRs have also been described in frogs [6]. Not only are these rogue RyRs situated far from the jSR, effectively ruling out any other possibility than regulation by CICR, but they also appear to be a different RyR isoform (RyRβ) compared to those in the jSR membrane (RyRα; [1]). The expression ratio of these two calcium release channels was found to be 1:1 [7].

Not only do amphibian skeletal muscles differ from those in mammals in their RyR composition and localization—mammals have only a single RyR isoform in most of their adult skeletal muscles (RyR1; [8])—but the functional appearance of calcium release is also different. While elementary calcium release events (ECREs) are difficult to identify in mammals—they are small-amplitude, long-lasting increases in calcium concentration [9]—amphibian muscles display calcium sparks—ECREs that are short and relatively large in amplitude—involving the simultaneous opening of several calcium release channels [9] and, probably, the operation of CICR. These functional differences are likely related to the presence and distribution of RyR, since the expression (using injected plasmid) of RyR3—the embryonic mammalian RyR isoform resembling RyRβ in its calcium sensitivity—in adult mouse skeletal muscle led to the appearance of calcium sparks [10,11]. Neonatal mammalian skeletal muscle contains both RyR1 and RyR3 in equal amounts [12] and lacks a well-developed T-tubule system. The acceleration of CICR by RyR3 seems to ensure a uniform and synchronous activation of calcium release throughout the neonatal cell [13].

There are competing hypotheses about the number of ryanodine receptors (RyRs) involved in generating a calcium spark. Some studies suggest that a single RyR might be enough [14,15], while others propose that either the entire RyR release unit [16,17] or only a subset of RyRs within the unit are activated [18,19,20]. Wang et al. [20] demonstrated that the local Ca^2+^ signal caused by a single opening of a DHPR (Ca^2+^ sparklet) can activate about 4–6 RyRs, leading to a calcium spark in cardiac myocytes. The exponential distribution of the coupling latency suggests that the connection between DHPRs and RyRs is random. Additionally, not all sparklets can successfully trigger a spark. The authors presented a sparklet-triggered spark and its temporal profile, which shows a visible flaw during the rising phase.

In our previous study [21], we conducted a detailed analysis of the morphology and sarcomeric localization of calcium sparks in two-dimensional images and proposed that ryanodine receptors (RyRs) may open sequentially. This was already suggested by Zhou et al. [22]: the sequential propagation of calcium release from the SR requires parajunctional channels that are present only in frogs. To further test this hypothesis, we now examine the effects of membrane depolarization and caffeine on spark parameters, morphology, and spatial distribution using high-resolution line-scan imaging. While membrane depolarization is expected to primarily affect RyRs located in the junctional sarcoplasmic reticulum, caffeine, by increasing the sensitivity to calcium-induced calcium release, is more likely to modulate parajunctional calcium-release channels. Consistent with this, we find that caffeine alters a greater number of spark parameters than depolarization. Moreover, using of ultra-high-speed acquisition, we provide evidence that calcium sparks can, indeed, arise from the sequential activation of individual release channels or channel clusters.

## 2. Materials and Methods

### 2.1. Fiber Preparation and Solutions

American bullfrogs (*Rana pipiens*) were placed in a slurry of crushed ice and water for 30 min, then euthanized by decapitation followed by spinal cord destruction, following protocols approved by the University of Maryland Institutional Animal Care and Use Committee. Toe muscles (*flexor digitorum brevis*) were dissected, and individual muscle fibers were freshly enzymatically isolated on the day of experiments using 8 mg/mL neutral dispase II and 1.3 mg/mL collagenase A at 37 °C for 1 h in a nominally calcium-free Ringer solution (in mM: 115 NaCl, 2.5 KCl, 1 MgCl_2_, 10 HEPES, pH 7.0). The intact, isolated fibers were plated onto glass-bottom dishes coated with extracellular matrix in Ringer solution containing 1.8 mM CaCl_2_. Before each experiment, cells were incubated with 20 µM Fluo-4 AM at room temperature for 30 min, followed by a 20 min de-esterification period. To evoke voltage-dependent calcium sparks in intact fibers, extracellular solutions with elevated potassium ion (K^+^) concentrations were used (in mM: 115 NaCl, 1 MgCl_2_, 1.8 CaCl_2_, 10 HEPES, 13.8 K methane sulfonate, pH 7.0) without triggering transient rises in intracellular calcium or muscle contractions. The Goldman–Hodgkin–Katz equation was used to calculate the actual membrane potential, and methanesulfonic acid was used to maintain a constant Cl^−^ concentration, ensuring consistent ionic strength and osmolality [21]. To enhance the frequency of calcium sparks, 1 mM caffeine was added. Individual muscle fibers were monitored, and those exhibiting increased global fluorescence or physical deformation following laser exposure or solution changes were excluded from spark recordings. Baseline spontaneous activity was recorded in fibers before applying mild depolarizations or 1 mM caffeine. All precautions were taken to avoid photodamage and photobleaching, and subsequent images were acquired from the same fiber after repositioning. To minimize motion artefacts, local perfusion was not used during recordings.

### 2.2. High-Speed Recording and Analysis of Ca^2+^ Sparks in Intact Fibers

Fibers were scanned using a Zeiss LSM 5 LIVE laser scanning confocal microscope (Zeiss, Oberkochen, Germany). This system scans entire lines simultaneously rather than pixel by pixel, allowing for high temporal resolution and fast image acquisition. Line-scan images were acquired at 15 µs and 50 µs speeds, with line (pixel) acquisition times of 15.4 and 47.4 µs. Fluo-4 was excited using a 488 nm argon-ion laser, and emitted light was collected through a beam-path filter and digitized at 12-bit resolution (pinhole size: 17 µm; objective: 63× water immersion, NA: 1.2). In the control (normal Ringer) condition, 191 line-scan images were recorded on 14 muscle fibers from 8 animals; in the presence of 1 mM caffeine, 545 line-scan images were recorded on 27 muscle fibers from 11 animals; at subthreshold depolarization, 419 line-scan images were recorded on 27 muscle fibers from 14 animals. The different conditions were investigated separately. For each experiment, a new Petri dish with 1 mL of cell suspension was used. In each Petri dish, a certain condition was used; the healthy-looking fibers (1–6) were imaged in a way that each line scan was recorded at different positions to avoid photobleaching. Damaged and/or contracting muscle fibers were excluded from experiments.

Line-scan Analysis. Images were normalized using standard procedures described by Cheng et al. [15]. Spark detection was performed using a threshold-based algorithm, applying levels 8 and 9 of the one-dimensional à trous wavelet transform along the time axis, similar to the calcium ember detection method reported in Szabó et al. [23]. Spark parameter analysis was conducted within the detected region, which was extended by 100 pixels in the time (t) direction before and after the spark and by 20 pixels in the spatial (x) direction. The amplitude (A) and full width at half maximum (FWHM) were calculated at each time point within the selected region by fitting a Gaussian function to the spatial fluorescence profile, following the method of Hollingworth et al. [24]. For each time point, data for the fit were obtained by averaging values from 9 adjacent scan lines. If the Gaussian fit failed to converge, A and FWHM were assigned a value of 0. This procedure generated two discrete time series—amplitude (called Gaussian-fitted) and FWHM—corresponding to each event, defined only at time points where the least-squares Gaussian fitting successfully converged. In addition, an estimated amplitude was also calculated by sliding a 7 × 5 pixel window (time × space) along the central time axis of the spark and averaging the 9 highest-intensity pixels within each window. This estimated amplitude value (called averaged) was defined at all time points, regardless of fit convergence. Spark duration was defined as the time difference t_2_ − t_1_, where t_1_ is the time point at which the estimated amplitude first exceeds 10% of its peak value, with no subsequent drop below that threshold, and t_2_ is the first time point after the peak when the descending estimated amplitude reaches 15% of the peak value. The rise time of the spark was calculated as the difference t_peak_ − t_1_. The histograms of spark parameters were fitted with the Lognormal function:

(1) $$y=\frac{A}{X}e^{\left[-0.5{\left(\frac{ln(\frac{X}{X_{0}})}{B}\right)}^{2}\right]},$$

X_0_ is the expected value or mean, B is the SD, and A is a scaling factor.

### 2.3. Signal Mass Parameter Calculation

Signal mass (SM) was calculated as follows:

(2) $$SM=1.206\times Amp\times {FWHM}^{3},$$

where Amp is the amplitude, while FWHM has its usual meaning [24]. Signal mass parameters (maximum and slope) were calculated beginning from the time point ts_1_, determined based on the following two criteria:

(a) The estimated amplitude reached at least 20% of its maximum value;

(b) Both amplitude (Amp) and FWHM values were available for all subsequent time points up to the signal peak (t_peak_).

Calculations were carried out up to the time point ts_2_, defined as 1 ms after t_peak_. The maximum signal mass was defined as the peak value of the signal mass curve between ts_1_ and ts_2_. The signal mass slope was determined by fitting a linear regression to the signal mass curve within the same interval. Pearson’s correlation coefficient was computed to assess the quality of this fit. In case the SM was a continuous curve in the interval between ts_1_ and ts_2_, then its first-order time derivative was also calculated. The time derivative of the calcium spark signal mass is proportional to the net calcium release flux or calcium release current flowing through the open ryanodine receptor channels at that specific moment.

### 2.4. Analysis of the Spark’s Time Course

Some sparks recorded with 15 or 50 µs/line were analyzed manually. A histogram of the Amp(t) function was created using a 0.015 F/F_0_ bin (0.02 and 0.025 F/F_0_ were also tested; see Figure S1) for each analyzed event. These histograms were then fitted with the sum of Gaussian functions. The best fit to the data points was accepted, and the peak positions of the individual Gauss functions were used to determine the locations of the fluorescence steps.

### 2.5. Chemicals and Statistical Analysis

Chemicals, unless otherwise stated, were purchased from Sigma (St. Louis, CA, USA) and were of analytical grade. Pooled data are expressed as the mean ± standard error (SE) of the mean. The differences between control and treatments were assessed using one-way analysis of variance (ANOVA) and all pairwise multiple comparison procedures (Student–Newman–Keuls Method). The F-test was used to test the significance, and a *p*-value of less than 0.05 was considered statistically significant. Statistics were performed with Graphpad Prism 6 (GraphPad Software, San Diego, CA, USA).

## 3. Results

### 3.1. The Effects of Depolarization and Caffeine on Spark Parameters

**Figure 1:**
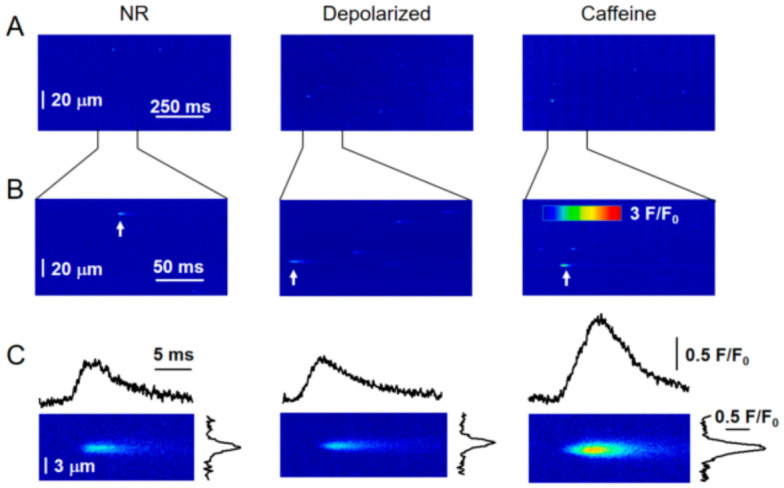
Sparks under control conditions, after subthreshold depolarization, and in the presence of caffeine: (**A**) 1 s-long line-scan images; (**B**) 200 ms-long line-scan images from images in panel A; (**C**) individual sparks with a temporal and spatial profile. Left column: control (normal Ringer, NR). Middle column: subthreshold depolarization to −65 mV. Right column: in the presence of 1 mM caffeine. Scales in the left column correspond to all images to the right. The color scale in the right column in the second row corresponds to all images. White arrows in panel B point to the spark shown in panel C. Scales for the temporal and spatial profiles in panel C correspond to all sparks.

**Figure 2:**
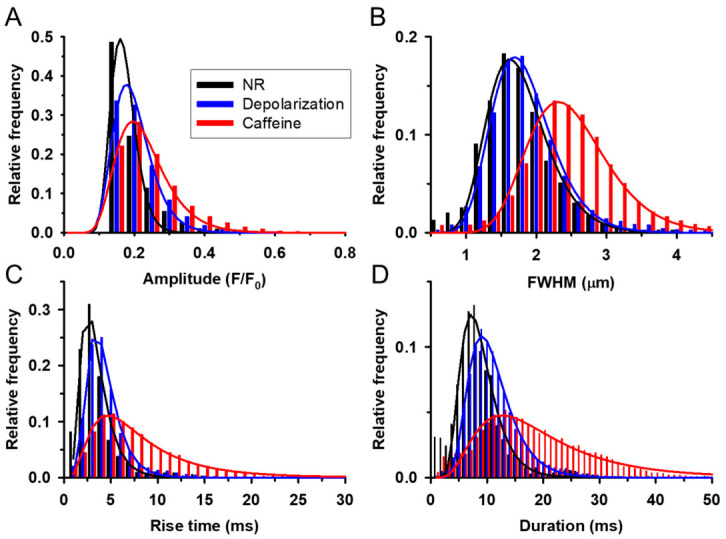
Histogram of spark parameters. Histograms of amplitude (**A**), FWHM (**B**), duration (**C**), and rise-time (**D**) of sparks in NR (black), subthreshold depolarization to −65 mV (blue), and in the presence of 1 mM caffeine (red). Solid curves represent the best fit of a Lognormal function to the points. The parameters of the fits are presented in Table S2.

Conventional confocal microscopes usually use a 1 ms/line scanning speed for line-scan measurements. In our experiments, we employed acquisition speeds of 50 and 15 μs/line, which are 20 and 66 times faster, respectively. Figure 1 shows representative line scans recorded at 50 μs/line under control conditions, after subthreshold depolarization to −65 mV, and in the presence of 1 mM caffeine. Panel A displays 1 s-long line scans, while panel B presents parts of images in panel A, which are five times more detailed. Finally, panel C illustrates single sparks across all conditions with their spatial and temporal profiles. We want to highlight the unprecedented detail captured in the sparks’ time profiles. Both depolarization and caffeine increased the frequency, amplitude, FWHM, rise time, and duration of sparks in frog skeletal muscle fibers (Table 1, Figure 1). The histograms of spark parameters clearly show that depolarization caused only a slight rightward shift in their distribution (Figure 2), whereas caffeine caused a significant rightward shift, mainly because of the high number of large-amplitude, wider sparks observed with the drug. The histograms were fitted with a Lognormal function. The results of the fits are presented in Table 1 and Table S1 and are shown on the histograms in Figure 2.

**Figure 3:**
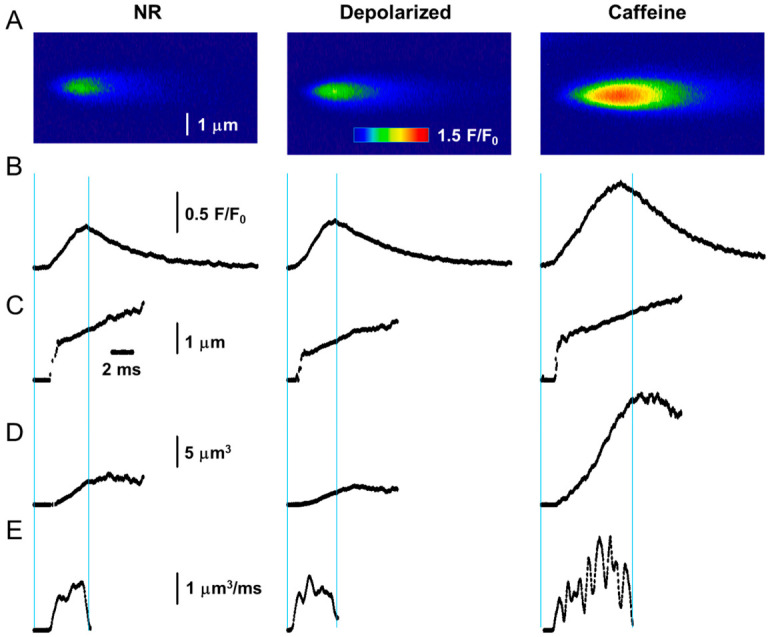
(**A**) Averaged sparks. (**B**) Amplitude profiles. (**C**) Time courses of FWHM. (**D**) Time courses of SM. (**E**) Time courses of release flux. Left column: control (normal Ringer, NR). Middle column: subthreshold depolarization to −65 mV. Right column: in the presence of 1 mM caffeine. Scales in the left column correspond to all panels to the right. The color scale in the middle column in the first row corresponds to all images. The time derivation of SM(t) (release flux calculation) was performed in the interval between the two vertical blue lines.

In a subset of events (amplitude ≥ 0.3 and 0.3 ≤ FWHM ≤ 3.0), the signal mass was calculated (see Section 2, Eqn. 2). Since both depolarization and caffeine increased the FWHM compared to control sparks, SM was also significantly higher under these conditions (Table 2). We aimed to calculate the rate of calcium release during a spark. For this, 10 to 20 sparks with similar amplitudes were averaged across all conditions. These averaged sparks are shown in Figure 3A. Thanks to averaging, the temporal profiles of amplitude (Figure 3B) and FWHM (Figure 3C) were rendered relatively noise-free, making the signal mass very smooth across all cases (Figure 3D). This enabled the calculation and assessment of the time derivative of SM (the flux of calcium through RyRs) (Figure 3E). We applied these steps to high-amplitude individual sparks as well. If the derivative was calculable, the flux was computed (for subsets of events, see the criteria above) and averaged (Table 2). Interestingly, depolarization did not significantly increase the release flux, whereas caffeine could enhance the rate of calcium release. The histogram of SM (Figure S2A, Table S2) resembles the histograms of amplitude (Figure 2A) and FWHM (Figure 2B), where caffeine caused a significant rightward shift, while depolarization did not (Figure S2B, Table S2).

### 3.2. Detailed Analysis of the Temporal Profile of Spark Amplitude

**Figure 4:**
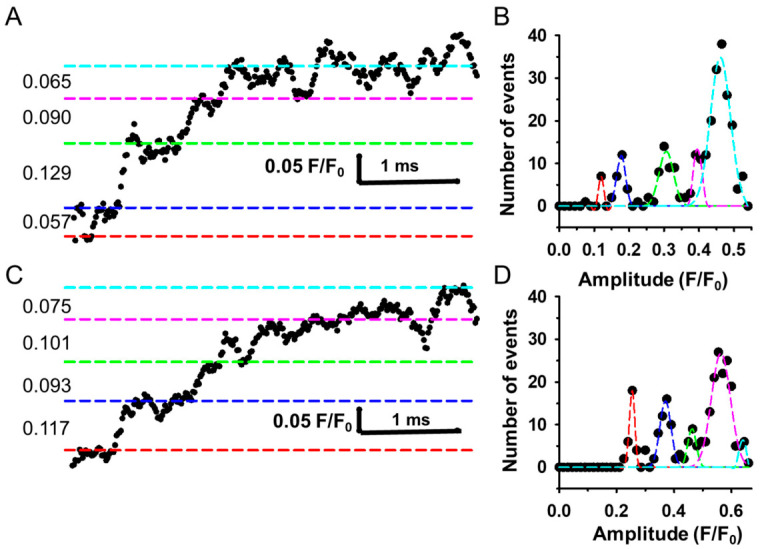
Step size analysis of the rising phase from two sparks with a bin of 0.015 F/F_0_. (**A**,**C**) Temporal profile of the amplitude of a spark. Dashed horizontal lines show the steps calculated as the position of peaks of the color-matched Gaussian function in panels B and D. Numbers on the left side show the distance between steps in F/F_0_. (**B**,**D**) Histogram created from the rising phase and shows the distribution of normalized fluorescence with a bin of 0.015 F/F_0_. Note the clear separation of the individual Gaussians. Colored dashed lines show the fitted five Gaussian functions (red, blue, green, magenta, and cyan).

We were surprised to find that the derivative of SM over time is not a smooth, increasing function during spark activation; instead, it exhibits breaks (Figure 3E). This observation prompted a detailed analysis of spark amplitude using high-speed line scans (15 μs/line). To identify visible steps in the sparks’ temporal profile (Figure 4A,C and Figure S1A), we generated histograms of normalized fluorescence intensities, assuming that their peaks represent distinct fluorescence levels. We tested bin width of 0.15, 0.20, and 0.25 F/F_0_. As shown in Figure S1B, 0.15 F/F_0_ proved to be the optimal bin width. A bin of 0.20 F/F_0_ resulted in a less wavy curve (Figure S1C), while 0.25 F/F_0_ produced a curve with too few points for reliable fitting (Figure S1D). After fixing the 0.15 F/F_0_ bin (see also the justification of the noise analysis at the end of the Results section), we performed fits of the activation phase (from start to peak, Figure 4A,C). To identify the number and position of peaks, we fitted a sum of Gaussian functions to the data points. The best fits are displayed in Figure 4B,D and Figure S1B–D. When examining the activation phases and isolating the individual steps, it became clear that, in certain cases, as indicated by the larger difference in the positions of two consecutive steps (0.129 in Figure 4A), a merging of two steps was observed.

**Figure 5:**
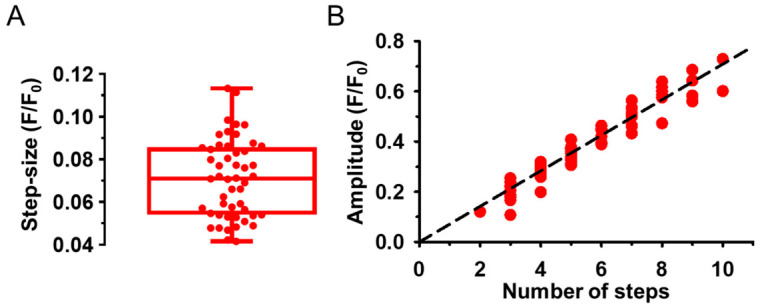
Step size analysis of sparks’ amplitude determined with Gaussian fitting. (**A**) The points represent the step positions for the rising phase from the Gaussian fitted amplitude curve with a bin of 0.015 F/F_0_. The box plots present the average step size. (**B**) Dashed lines show the best fit of a y = m·x linear function to the points. The slope (m) is 0.071 ± 0.003 F/F_0_ (R^2^ = 0.925).

The detailed analysis included 15 sparks with varying amplitudes but clear steps in their temporal profiles. The number of steps derived from Gaussian fits (4.27 ± 0.45) was corrected for the presence of merging steps, giving rise to an average step size of 0.077 ± 0.03 F/F_0_ (Figure 5A). This analysis was performed on both the amplitude profile generated by averaging and by Gaussian fitting (see Section 2, Figure S3). In case of the averaged amplitude profile, the average number of fitted steps was 4.53 ± 0.38 and the average step size was 0.075 ± 0.004 F/F_0_ (Figure S5A). None of these differed significantly from the values obtained from the Gaussian fitted activation phase. Lastly, we examined how the number of steps relates to the spark’s amplitude. Figure S4 shows a low-and a high-amplitude spark with its steps. The low-amplitude spark analysis identified two steps, whereas the high-amplitude spark analysis found five. As expected, a strong linear relationship exists between the amplitude and the number of steps, regardless of whether the amplitude profile was obtained via Gaussian fitting (Figure 5B) or averaging (Figure S4B). The linear fits yielded slopes of 0.071 ± 0.003 F/F_0_ (Figure 5B, R^2^ = 0.925) for Gaussian fitting and 0.073 ± 0.001 F/F_0_ (Figure S4D, R^2^ = 0.85) for the averaged amplitude profile, matching the step size results.

To ensure that the calculated step sizes were not artifacts of noise, we also examined the distribution of fluorescence values in spark-free regions, representing background noise. In these regions, the histograms could be fitted with single Gaussian functions; an example is shown in Figure S6. The average standard deviation obtained from these fits was 0.0346 ± 0.0020 F/F_0_. According to Scott’s normal-reference rule [25], the optimal bin width is given by 3.49σn^−1/3^, where *σ* is the standard deviation of the baseline noise and *n* is the number of data points in the sample. For our records, this corresponds to a bin width of approximately 0.5σ, or about 0.015 F/F_0_, which was used to generate the data shown in Figure 4 and Figure 5. To assess the robustness of the obtained step size, the binning analysis was repeated using bin widths of 0.020 and 0.025 F/F_0_. The corresponding step sizes were 0.0748 ± 0.0034 F/F_0_ and 0.0816 ± 0.0035 F/F_0_, respectively. These results were not significantly different from which was obtained with bin width of 0.015 F/F_0_ (*p* > 0.5), and indicate that the presence of the steps is largely independent of the chosen bin width and that the estimated step size is robust (Figure S7). Nevertheless, we emphasize that the absolute value of the step size depends on the experimental conditions, including the choice of dye and the confocal system used, and should, therefore, be interpreted with these factors in mind.

## 4. Discussion

In this study, we provide an in-depth analysis of elementary calcium release events (Ca^2+^ sparks) in intact frog skeletal muscle fibers using ultra-high-speed line-scan confocal microscopy. We compare sparks induced by mild depolarization and those evoked by caffeine, revealing clear differences in frequency, amplitude, duration, and spatial extent. Importantly, through high temporal resolution imaging (15 or 50 µs per line), our study identifies stepwise transitions in fluorescence during the activation phases of individual sparks. These findings strongly suggest that Ca^2+^ sparks result from the sequential activation of discrete RyR groups, rather than a purely stochastic or all-or-none opening of a single release unit. This work significantly advances our understanding of excitation–contraction (E–C) coupling in amphibian skeletal muscle and provides mechanistic insight into how neighboring RyR channels may coordinate during local calcium release events.

### 4.1. Comparison of Depolarization- and Caffeine-Induced Sparks

**Figure 6:**
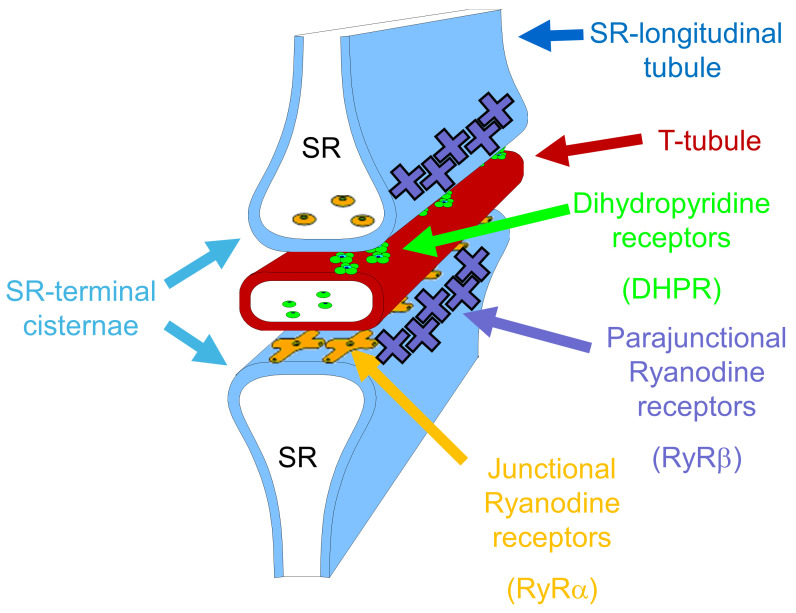
Triad model for a frog skeletal muscle with two RyR isoforms. A double row of orthogonally placed junctional RyRs (orange) occupies the gap between the T-tubule and SR. Parajunctional RyRs (blue) are located in the adjacent SR membrane. They are arranged in a zig-zag pattern. Modified from [6].

Our results clearly demonstrate that both subthreshold depolarization (to –65 mV) and caffeine (1 mM) increase the frequency of Ca^2+^ sparks compared to control conditions, though the underlying mechanisms differ. Depolarization activates RyRs indirectly via DHPR voltage sensors localized to junctional SR regions, while caffeine sensitizes RyRs in the SR membrane by facilitating CICR. Under depolarization, spark amplitude decreased slightly, suggesting that the DHPR-mediated activation of RyRs is spatially restricted to the junctional RyRα isoform population and may involve fewer open channels per event. In contrast, caffeine substantially increased amplitude, duration, FWHM, and signal mass. These effects likely reflect enhanced recruitment of parajunctional or “rogue” RyRs (RyRβ isoform) that are not physically coupled to DHPRs but are more sensitive to cytosolic Ca^2+^. This differential modulation highlights the compartmentalized control of calcium signaling in amphibian muscle: DHPR-linked RyRs respond to voltage, whereas parajunctional RyRs amplify release via CICR once the local Ca^2+^ concentration surpasses the threshold (Figure 6). In separate experiments [21], we found that caffeine and depolarization had additive effects on spark frequency, supporting the notion of distinct, yet interacting RyR populations contributing to total calcium release.

### 4.2. Morphological Characteristics and Kinetic Properties of Sparks

Quantitative analysis revealed that caffeine-induced sparks were larger, longer-lasting, and spatially broader than those produced by depolarization. Specifically, FWHM increased from ~1.8 μm in depolarized cells to ~2.2 μm with caffeine, and duration nearly doubled from ~10.8 ms to ~17.3 ms. The rise time also increased proportionally (from 4.1 ms to 7.3 ms), suggesting slower recruitment and termination kinetics. These morphological differences are consistent with an expanded Ca^2+^ diffusion and prolonged channel opening times under caffeine. Such kinetic alterations imply that CICR-driven sparks may involve not only more channels but also a less synchronized gating pattern, possibly reflecting sequential recruitment of neighboring release units.

### 4.3. Signal Mass and Calcium Release Flux

By calculating the signal mass (SM) from spark amplitude and FWHM (Eqn. 2), the total calcium released during each event was estimated [24]. Caffeine substantially increased SM, whereas depolarization alone had little effect. Interestingly, while caffeine enhanced total release, the release flux—the derivative of signal mass over time—did not scale proportionally. This finding suggests that caffeine primarily extends the duration rather than the instantaneous rate of calcium release. From a mechanistic perspective, this implies that caffeine can prolong RyR open time or may promote repeated reopenings within the same release event. The lack of a flux increase with depolarization implies that voltage activation recruits a fixed number of RyRs per event, limited by the geometric arrangement of DHPR–RyR couplings, without altering channel kinetics. These distinctions align with previous work, which shows that DHPR activation yields tightly synchronized, transient release, whereas CICR-based activation yields more variable, spatially diffuse events [22]. Taking the derivative of the signal mass, allows us to analyse the behavior of intracellular channels as if they were performing an electrical patch-clamp experiment on a single living cell compartment [26]. Thus, we can assume that the signal mass is proportional to the total quantity of Ca^2+^ released into the cytosol, and its rate of rise is proportional to the Ca^2+^ current flowing through the RyRs during a spark.

### 4.4. Discovery of Stepwise Activation

The most striking result of the study is the identification of stepwise transitions in fluorescence intensity during the rise of individual Ca^2+^ sparks. Using 15 µs line-scan resolution, we observed discrete steps in normalized fluorescence (F/F_0_), typically with an average step size of ~0.07 F/F_0_. This quantization is a critical observation, as it implies that RyR gating within a release unit is neither perfectly synchronized nor random but proceeds in sequential subunits of fixed fluorescence contribution. Each step likely represents the coordinated opening or closing of a small group of RyRs—perhaps a single cluster within a couplon or a small group of parajunctional channels triggered by local Ca^2+^ elevation. The strong linear correlation between the number of steps and total spark amplitude indicates that total fluorescence is proportional to the number of recruited units. This supports a model where calcium sparks are the summation of discrete, quantal subevents, each arising from a limited number of RyR channels.

### 4.5. Physiological Interpretation: Sequential Recruitment Model

We interpret these findings as evidence that calcium release during a spark proceeds by sequential activation of neighboring RyR channels. This model integrates several previous hypotheses: (1) CICR-mediated propagation of local release, (2) coupled or allosteric gating among adjacent RyRs, and (3) structural heterogeneity within triadic junctions. In the amphibian system, RyRα and RyRβ isoforms coexist, with the latter positioned parajunctionally and more responsive to cytosolic calcium. Depolarization first activates RyRα via mechanical coupling to DHPRs, generating a small initial Ca^2+^ flux. This local increase in Ca^2+^ then triggers adjacent RyRβ clusters through CICR, creating a cascade of activation steps that manifest as the observed fluorescence increments. The relatively constant step size across events implies that each recruited cluster contributes a fixed quantum of calcium flux. The linear relation between spark amplitude and the number of steps supports this modular assembly of release units. In this sense, sparks can be viewed as microdomains of graded recruitment, rather than binary all-or-none responses. This interpretation reconciles earlier controversies regarding the number of RyRs required for a spark. Instead of a single channel (as proposed by Cheng et al. [14]) or all channels within a couplon [16], the data suggest intermediate recruitment: each spark represents the cumulative contribution of several RyR channels, each opening in sequence, and contributing a quantal step to the total fluorescence signal. Sometimes, however, two RyRs can open nearly simultaneously, producing a step in the fluorescence record that is twice as large as would be expected from the opening of a single RyR.

### 4.6. Comparison with Previous Results and Models

Earlier studies in cardiac and skeletal muscle have provided indirect hints of quantal behavior in Ca^2+^ sparks, but none had achieved the temporal resolution necessary to resolve discrete steps in vivo. The 15 µs/line acquisition used here represents a technological leap, enabling direct visualization of rapid transitions that would otherwise be averaged out in slower scans (~1 ms/line typical of conventional confocal microscopic systems). The observed stepwise pattern in this study matches findings in cardiac myocytes, where “couplons” of 4–6 RyRs were proposed to open stochastically but with partial synchrony [17]. A similar result was found in cardiac myocytes by Shkry et al. [27]. The authors analyzed ultra-fast confocal line scans and found discrete steps in the activation phase of the sparks. In frog muscle, the spatial organization of RyRs—double rows with alternating DHPR coupling—naturally lends itself to sequential activation. The presence of parajunctional RyRβ further facilitates local CICR propagation, as previously hypothesized by Zhou et al. [22] and demonstrated morphologically by Felder & Franzini-Armstrong [6]. The quantal fluorescence increments observed here resemble the “calcium step” or “sparklet” phenomena described in cardiac [28] and smooth muscle [29], though the context differs. In cardiac cells, stepwise increases in calcium current reflect stochastic DHPR openings; in the current work, they arise from RyR clusters.

The differential effects of membrane depolarization and the application of caffeine were also studied earlier. Kashiyama and coworkers [30] expressed RyRα and RyRβ in RyR-deficient myotubes. RyRα but not RyRβ exhibited Ca^2+^ transients triggered by membrane depolarization. They observed RyRα mediated graded and sustained uniform Ca^2+^ release throughout the cytoplasm, whereas RyRβ initiated all-or-none-type regenerative calcium oscillations and waves. The authors hypothesized that these distinct properties may also occur in frog skeletal muscle.

### 4.7. Implications for Muscle Physiology

Although the study is performed on amphibian muscle, the findings have broader implications for excitation–contraction coupling across species. In adult mammalian muscles where RyR3 is absent [12]—with the exception of the diaphragm (~13%) [12,31] and the extraocular muscles [32]—and only RyR1 exists, classical calcium sparks are rare and of smaller amplitude. Introducing RyR3 into mammalian muscle [10,11] restores spark-like behavior, supporting the idea that the presence of a secondary Ca^2+^-sensitive RyR population is essential for stepwise release. Thus, the frog muscle model represents a natural hybrid system combining both voltage-controlled and CICR-sensitive RyRs, providing insight into how these two modalities coexist and interact in vivo. The sequential gating model suggests a mechanism for amplifying small depolarization signals into robust local release events, ensuring reliable E–C coupling while avoiding uncontrolled calcium waves. Furthermore, quantized calcium release could play a role in modulating graded contractile responses. By adjusting the number of recruited RyR groups per event, muscle fibers may fine-tune local calcium levels and, consequently, the activation of nearby contractile elements. This graded control could be particularly important during subthreshold stimuli or fatigue conditions, where precise regulation of calcium release prevents unnecessary energy expenditure or calcium overload.

### 4.8. Integration with Broader Theoretical Models

The sequential gating mechanism observed here aligns with the “local control” theory of E–C coupling, which posits that each DHPR–RyR cluster functions as a semi-independent release unit [33]. The current findings refine this model by adding a temporal dimension: Within each unit, subgroups of RyRs open successively, leading to quantal fluorescence steps. This challenges the binary view of couplon activation and introduces a graded model of local release. From a systems perspective, such quantization may confer stability to calcium signaling networks. Stepwise recruitment ensures that local calcium release remains spatially confined, preventing runaway CICR that could trigger global calcium waves or contracture. It also allows fine-tuned modulation of release strength, balancing sensitivity and robustness. Mathematical modeling of such behavior could involve Markov or cooperative binding models, where each RyR group transitions between closed and open states with rates influenced by local Ca^2+^. Incorporating experimentally measured step sizes and durations could constrain these models, providing quantitative predictions for spark variability under different physiological conditions.

### 4.9. Limitations and Future Directions

While the study provides compelling evidence for stepwise RyR activation, several questions remain open. First, the exact structural identity of the channel groups corresponding to each fluorescence step is not directly observed. Correlative imaging techniques, such as electron tomography combined with functional mapping, would be valuable for linking quantal steps to specific RyR clusters. Second, while caffeine and depolarization were used to bias RyR activation, other modulators—such as Mg^2+^, ATP, or pharmacological RyR stabilizers—could be tested to explore how channel cooperativity changes with physiological state. Extending this approach to mammalian fibers expressing both RyR1 and RyR3 might further clarify the role of isoform diversity in shaping release quantization.

## 5. Conclusions

Our study provides compelling experimental evidence that calcium sparks in frog skeletal muscle fibers arise from the stepwise activation of discrete groups of RyR channel. Using unprecedented temporal resolution, we reveal quantized fluorescence transitions that directly reflect sequential channel gating. Caffeine and depolarization can modulate spark properties in distinct but complementary ways, consistent with the involvement of separate RyR populations (junctional and parajunctional). The constant step size (~0.07 F/F_0_) and linear relationship between amplitude and step number demonstrate that each quantal increment corresponds to a fixed unit of calcium release, likely representing a small RyR cluster. These findings unify structural and functional observations of RyR organization, supporting a model in which local calcium release is graded, modular, and dynamically coordinated.

Beyond its relevance to amphibian muscle physiology, this work lays a conceptual and methodological foundation for future exploration of quantal calcium signaling in other excitable cells. By bridging molecular structure, channel kinetics, and optical physiology, our study advances our understanding of how microscopic channel interactions give rise to macroscopic muscle function. Understanding the role of CICR in generating stepwise calcium sparks may help reveal the mechanisms behind these events during pathological conditions and regeneration in mammalian skeletal muscle. Additionally, the roles of amphibian RyRα and RyRβ in producing spontaneous calcium sparks should provide insight into how RyR1 and RyR3 interact in mammalian muscle fibers where RyR3 is present.

## Figures and Tables

**Table 1:** Parameters of sparks.

	NR	Depolarized	Caffeine
Number of line scans	191	419	545
Number of events	1023	16,316	19,196
Frequency (Hz/mm)	50.94	368.83 *	491.38 *^#^
Amplitude (F/F_0_)	0.167 ± 0.002	0.192 ± 0.002 *	0.218 ± 0.002 *^#^
FWHM (μm)	1.724 ± 0.017	1.793 ± 0.008 *	2.447 ± 0.012 *^#^
Rise time (ms)	3.100 ± 0.067	3.966 ± 0.036 *	6.913 ± 0.141 *^#^
Duration (ms)	8.241 ± 0.119	10.340 ± 0.049 *	17.440 ± 0.370 *^#^

* Indicates a significant difference from control at *p* < 0.001; ^#^ indicates a significant difference from depolarized at *p* < 0.001. Only events with an amplitude equal to or greater than 0.1 F/F_0_ were included in the analysis.

**Table 2:** Parameters of signal mass of sparks.

	NR	Depolarized	Caffeine
Number of events	25	230	858
SM maximum (μm^3^)	3.551 ± 0.403	4.190 ± 0.135	8.659 ± 0.160 *^#^
dSM/dt (μm^3^/ms)	0.861 ± 0.077	0.88 ± 0.028	1.033 ± 0.017 ^#^

* Indicates a significant difference from control at *p* < 0.001; ^#^ indicates a significant difference from depolarized at *p* < 0.001. Only events with an amplitude higher than 0.3 F/F_0_ and a FWHM between 0.3 and 3.0 µm were included in the analysis.

## Data Availability

The raw data supporting the article’s conclusions will be made available by the authors upon reasonable request.

## Supplementary Materials

The following supporting information can be downloaded at: https://www.mdpi.com/article/10.3390/biom16060910/s1, Figure S1: Histogram of A(t) created with different bins; Figure S2: Histogram of signal mass and release flux; Figure S3: Comparison of the step size analysis of a spark determined with Gaussian fitting or averaging; Figure S4: Analysis of a low and a high amplitude spark; Figure S5: Step size analysis of sparks’ amplitude determined with averaging; Figure S6: Analysis of the background; Figure S7: The effects of the bin width on the step size; Table S1: Parameters of Log-Normal fits to histograms in Figure 2; Table S2: Parameters of Log-Normal fits to histograms in Figure S2.

## Abbreviations

The following abbreviations are used in this manuscript:

CICR	calcium-induced calcium release
DHPR	dihydropyridine receptor
ECRE	elementary calcium release event
FWHM	full width at half maximum
jSR	junctional sarcoplasmic reticulum
NA	numerical aperture
RyR	ryanodine receptor
SM	signal mass
SR	sarcoplasmic reticulum

## References

[1] Franzini-Armstrong C., Protasi F. (1997). Ryanodine Receptors of Striated Muscles: A Complex Channel Capable of Multiple Interactions. Physiol. Rev..

[2] Yin C.C., D’Cruz L.G., Lai F.A. (2008). Ryanodine Receptor Arrays: Not Just a Pretty Pattern?. Trends Cell Biol..

[3] Paolini C., Protasi F., Franzini-Armstrong C. (2004). The Relative Position of RyR Feet and DHPR Tetrads in Skeletal Muscle. J. Mol. Biol..

[4] Marx S.O., Ondrias K., Marks A.R. (1998). Coupled Gating between Individual Skeletal Muscle Ca^2+^ Release Channels (Ryanodine Receptors). Science.

[5] Ríos E., Pizarro G. (1991). Voltage Sensor of Excitation–Contraction Coupling in Skeletal Muscle. Physiol. Rev..

[6] Felder E., Franzini-Armstrong C. (2002). Type 3 Ryanodine Receptors of Skeletal Muscle Are Segregated in a Parajunctional Position. Proc. Natl. Acad. Sci. USA.

[7] Murayama T., Ogawa Y. (1992). Purification and Characterization of Two Ryanodine-Binding Protein Isoforms from Sarcoplasmic Reticulum of Bullfrog Skeletal Muscle. J. Biochem..

[8] Sorrentino V., Volpe P. (1993). Ryanodine Receptors: How Many, Where and Why?. Trends Pharmacol. Sci..

[9] Csernoch L. (2007). Sparks and Embers of Skeletal Muscle: The Exciting Events of Contractile Activation. Pflugers Arch. Eur. J. Physiol..

[10] Conklin M.W., Barone V., Sorrentino V., Coronado R. (1999). Contribution of Ryanodine Receptor Type 3 to Ca^2+^ Sparks in Embryonic Mouse Skeletal Muscle. Biophys. J..

[11] Pouvreau S., Royer L., Yi J., Brum G., Meissner G., Ríos E., Zhou J. (2007). Ca^2+^ Sparks Operated by Membrane Depolarization Require Isoform 3 Ryanodine Receptor Channels in Skeletal Muscle. Proc. Natl. Acad. Sci. USA.

[12] Flucher B.E., Conti A., Takeshima H., Sorrentino V. (1999). Type 3 and Type 1 Ryanodine Receptors Are Localized in Triads of the Same Mammalian Skeletal Muscle Fibers. J. Cell Biol..

[13] Yang D., Pan Z., Takeshima H., Wu C., Nagaraj R.Y., Ma J., Cheng H. (2001). RyR3 Amplifies RyR1-Mediated Ca^2+^-Induced Ca^2+^ Release in Neonatal Mammalian Skeletal Muscle. J. Biol. Chem..

[14] Cheng H., Lederer W.J., Cannell M.B. (1993). Calcium Sparks: Elementary Events Underlying Excitation-Contraction Coupling in Heart Muscle. Science.

[15] Cheng H., Song L.S., Shirokova N., González A., Lakatta E.G., Ríos E., Stern M.D. (1999). Amplitude Distribution of Calcium Sparks in Confocal Images: Theory and Studies with an Automatic Detection Method. Biophys. J..

[16] Satoh H., Katoh H., Velez P., Fill M., Bers D.M. (1998). Bay K 8644 Increases Resting Ca^2+^ Spark Frequency in Ferret Ventricular Myocytes Independent of Ca Influx: Contrast with Caffeine and Ryanodine Effects. Circ. Res..

[17] Sobie E.A., Duly K.W., Cruz J.D.S., Lederer W.J., Jafri M.S. (2002). Termination of Cardiac Ca^2+^ Sparks: An Investigative Mathematical Model of Calcium-Induced Calcium Release. Biophys. J..

[18] Bridge J.H.B., Ershler P.B., Cannell M.B. (1999). Properties of Ca^2+^ Sparks Evoked by Action Potentials in Mouse Ventricular Myocytes. J. Physiol..

[19] Cannell M.B., Cheng H., Lederer W.J. (1995). The Control of Calcium Release in Heart Muscle. Science.

[20] Wang S.Q., Song L.S., Lakatta E.G., Cheng H. (2001). Ca^2+^ Signalling between Single L-Type Ca^2+^ Channels and Ryanodine Receptors in Heart Cells. Nature.

[21] Cserne Szappanos H., Vincze J., Bodnár D., Dienes B., Schneider M.F., Csernoch L., Szentesi P. (2020). High Time Resolution Analysis of Voltage-Dependent and Voltage-Independent Calcium Sparks in Frog Skeletal Muscle Fibers. Front. Physiol..

[22] Zhou J., Brum G., González A., Launikonis B.S., Stern M.D., Ríos E. (2005). Concerted vs. Sequential. Two Activation Patterns of Vast Arrays of Intracellular Ca^2+^ Channels in Muscle. J. Gen. Physiol..

[23] Szabó L.Z., Vincze J., Csernoch L., Szentesi P. (2010). Improved Spark and Ember Detection Using Stationary Wavelet Transforms. J. Theor. Biol..

[24] Hollingworth S., Peet J., Chandler W.K., Baylor S.M. (2001). Calcium Sparks in Intact Skeletal Muscle Fibers of the Frog. J. Gen. Physiol..

[25] Scott D.W. (1979). On Optimal and Data-Based Histograms. Biometrika.

[26] Zhuge R., Fogarty K.E., Tuft R.A., Lifshitz L.M., Sayar K., Walsh J.V. (2000). Dynamics of Signaling between Ca^2+^ Sparks and Ca^2+^-Activated K^+^ Channels Studied with a Novel Image-Based Method for Direct Intracellular Measurement of Ryanodine Receptor Ca^2+^ Current. J. Gen. Physiol..

[27] Shkryl V.M., Blatter L.A. (2013). Ca^2+^ Release Events in Cardiac Myocytes Up Close: Insights from Fast Confocal Imaging. PLoS ONE.

[28] Wang S.Q., Wei C., Zhao G., Brochet D.X.P., Shen J., Song L.S., Wang W., Yang D., Cheng H. (2004). Imaging Microdomain Ca^2+^ in Muscle Cells. Circ. Res..

[29] Santana L.F., Navedo M.F. (2009). Molecular and Biophysical Mechanisms of Ca^2+^ Sparklets in Smooth Muscle. J. Mol. Cell. Cardiol..

[30] Kashiyama T., Murayama T., Suzuki E., Allen P.D., Ogawa Y. (2010). Frog α- and β-Ryanodine Receptors Provide Distinct Intracellular Ca^2+^ Signals in a Myogenic Cell Line. PLoS ONE.

[31] Jeyakumar L.H., Copello J.A., O’Malley A.M., Wu G.M., Grassucci R., Wagenknecht T., Fleischer S. (1998). Purification and Characterization of Ryanodine Receptor 3 from Mammalian Tissue. J. Biol. Chem..

[32] Eckhardt J., Bachmann C., Sekulic-Jablanovic M., Enzmann V., Park K.H., Ma J., Takeshima H., Zorzato F., Treves S. (2019). Extraocular Muscle Function Is Impaired in Ryr3^−/−^ Mice. J. Gen. Physiol..

[33] Stern M.D., Song L.-S., Cheng H., Sham J.S.K., Yang H.T., Boheler K.R., Ríos E. (1999). Local Control Models of Cardiac Excitation–Contraction Coupling. J. Gen. Physiol..

