# Supplementary material for: Sequential Gating of Ryanodine Receptors Underlies the Development of Calcium Sparks in Frog Skeletal Muscle
Source: Biomolecules. 2026 Jun 19;16(6):910. doi: 10.3390/biom16060910 (PMC13297114; doi:10.3390/biom16060910)
Supplement: Supplementary file 1 [file biomolecules-16-00910-s001.zip › new_supplementary_figure_legends.pdf]

Figure S1. Histogram of A(t) created with different bins. (**A**) Temporal profile of the rising phase of a spark. (**B**) Bin=0.015 F/F_0. (**C**) Bin=0.020 F/F_0. (**D**) Bin=0.025 F/F_0. The lines in panel A show the position of the steps. The solid red lines were calculated with a bin of 0.015 F/F_0. The long-dashed green lines were calculated with a bin size of 0.020 F/F_0. The short-dashed blue lines were calculated with a bin of 0.025 F/F_0.

Figure S2. Histogram of signal mass and release flux. Histograms of signal mass of sparks (**A**), and release flux during sparks (**B**) in NR (black), after depolarization to -65 mV (blue), and in the presence of 1 mM caffeine (red). Solid curves represent the best fit of a Lognormal function to the points. The parameters of the fits are in Table 2.

Figure S3. Comparison of the step size analysis of a spark determined with Gaussian fitting or averaging. The maximum amplitude is 0.524 and 0.481 F/F_0 for the Gaussian fitted and averaged, respectively. The average step size is 0.067±0.010 and 0.070±0.006 F/F_0 ($p>0.78$) for Gaussian fitted and averaged, respectively

Figure S4. Analysis of a low and a high amplitude spark. (**A**) A low-amplitude spark. (**B**) Amplitude profile. The inset shows the distribution of normalized fluorescence with a bin of 0.015 F/F_0. The red and blue dashed lines show the fitted Gaussian functions. The dashed red and blue horizontal lines show the steps calculated as the position of peaks of the color-matched Gaussian function. (**C**) A high-amplitude spark. (**D**) Amplitude profile. The inset shows the distribution of normalized fluorescence with a bin of 0.015 F/F_0. Color dashed lines show the fitted Gaussian functions. Dashed horizontal lines show the steps calculated as the position of peaks of the color-matched Gaussian function.

Figure S5. Step size analysis of sparks' amplitude determined with averaging. The points represent the step positions from the averaged (**A**, **B**) amplitude curve with a bin of 0.015 F/F_0. The box plots present the average step size for the averaged amplitude (**A**). Dashed lines show the best fit of a y=mx linear function to the points. The slope (m) is 0.073±0.001 F/F_0 (R^2=0.85) for the averaged amplitudes (**B**).

Figure S6. Analysis of the background. (**A**) Background before a spark. (**B**) Amplitude profile. The inset shows the distribution of normalized fluorescence with a bin of 0.015 F/F_0. The red solid line shows the fitted Gaussian function. The dashed red horizontal line shows the position of the peak of the fitted Gaussian function. A short horizontal black line labelled with 0 beneath shows the zero level of the trace.

Figure S7. The effects of the bin width on the step size. The average step-size calculated with bin width 0.015 (red, n=50), 0.02 (green, n=44), and 0.025 (blue, n=38) F/F_0.
