# Supplementary material for: Sequential Gating of Ryanodine Receptors Underlies the Development of Calcium Sparks in Frog Skeletal Muscle
Source: Biomolecules. 2026 Jun 19;16(6):910. doi: 10.3390/biom16060910 (PMC13297114; doi:10.3390/biom16060910)
Supplement: Supplementary file 1 [file biomolecules-16-00910-s001.zip › Suppl_Tables.pdf]

Table S1. Parameters of Log-Normal fits to histograms in Fig. 2.

		**NR**			**Depolarized**			**Caffeine**		
	**Bin**	**A**	**B**	**X_0**	**A**	**B**	**X_0**	**A**	**B**	**X_0**
**Amplitude**	0.01 F/F_0	0.081±0.004	0.209±0.014	0.167±0.002	0.07±0.002	0.277±0.01	0.192±0.002	0.058±0.002	0.389±0.01	0.218±0.002
**FWHM**	0.05 µm	0.296±0.01	0.248±0.01	1.724±0.017	0.312±0.005	0.241±0.044	1.793±0.008	0.317±0.005	0.236±0.004	2.447±0.012
**Rise time**	1.00 ms	0.84±0.033	0.447±0.024	3.1±0.067	0.952±0.017	0.392±0.008	3.966±0.036	0.629±0.015	0.638±0.017	6.913±0.141
**Duration**	1.00 ms	0.963±0.031	0.371±0.014	8.241±0.119	1.049±0.011	0.364±0.004	10.34±0.049	0.707±0.02	0.565±0.019	17.44±0.37

[1] Note: X_0 is the expected value or mean, B is the SD, and A is a scaling factor. See Materials and Methods for Eqn. 1.

Table S2. Parameters of Log-Normal fits to histograms in Fig. S2.

		**NR**			**Depolarized**			**Caffeine**		
	**Bin**	**A**	**B**	**X_0**	**A**	**B**	**X_0**	**A**	**B**	**X_0**
**SM**	1.0 μm^3	0.768±0.153	0.533±0.123	3.694±0.507	0.914±0.031	0.448±0.018	4.46±0.087	0.731±0.021	0.563±0.019	8.29±0.18
**dSM/dt**	0.2 μm^3/ms	0.199±0.025	0.381±0.054	0.871±0.051	0.194±0.008	0.417±0.02	0.918±0.02	0.188±0.003	0.424±0.009	1.036±0.01

[2] Note: X_0 is the expected value or mean, B is the SD, and A is a scaling factor. See Materials and Methods for Eqn. 1.
