# Supplementary figures and images for: Sequential Gating of Ryanodine Receptors Underlies the Development of Calcium Sparks in Frog Skeletal Muscle
Source: Biomolecules. 2026 Jun 19;16(6):910. doi: 10.3390/biom16060910 (PMC13297114; doi:10.3390/biom16060910)

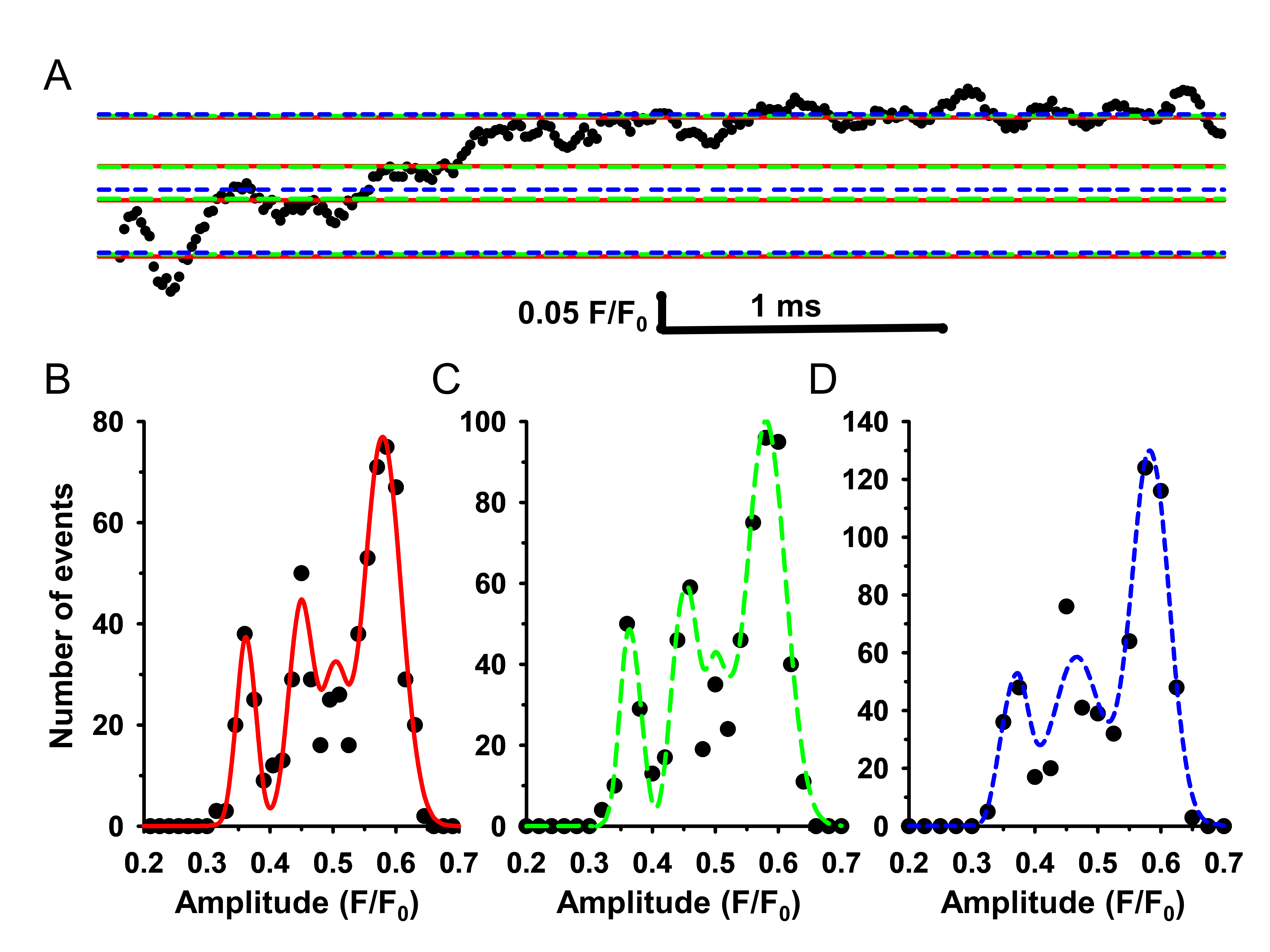

Supplement: Supplementary file 1 [file biomolecules-16-00910-s001.zip › Figure_S1.tif]

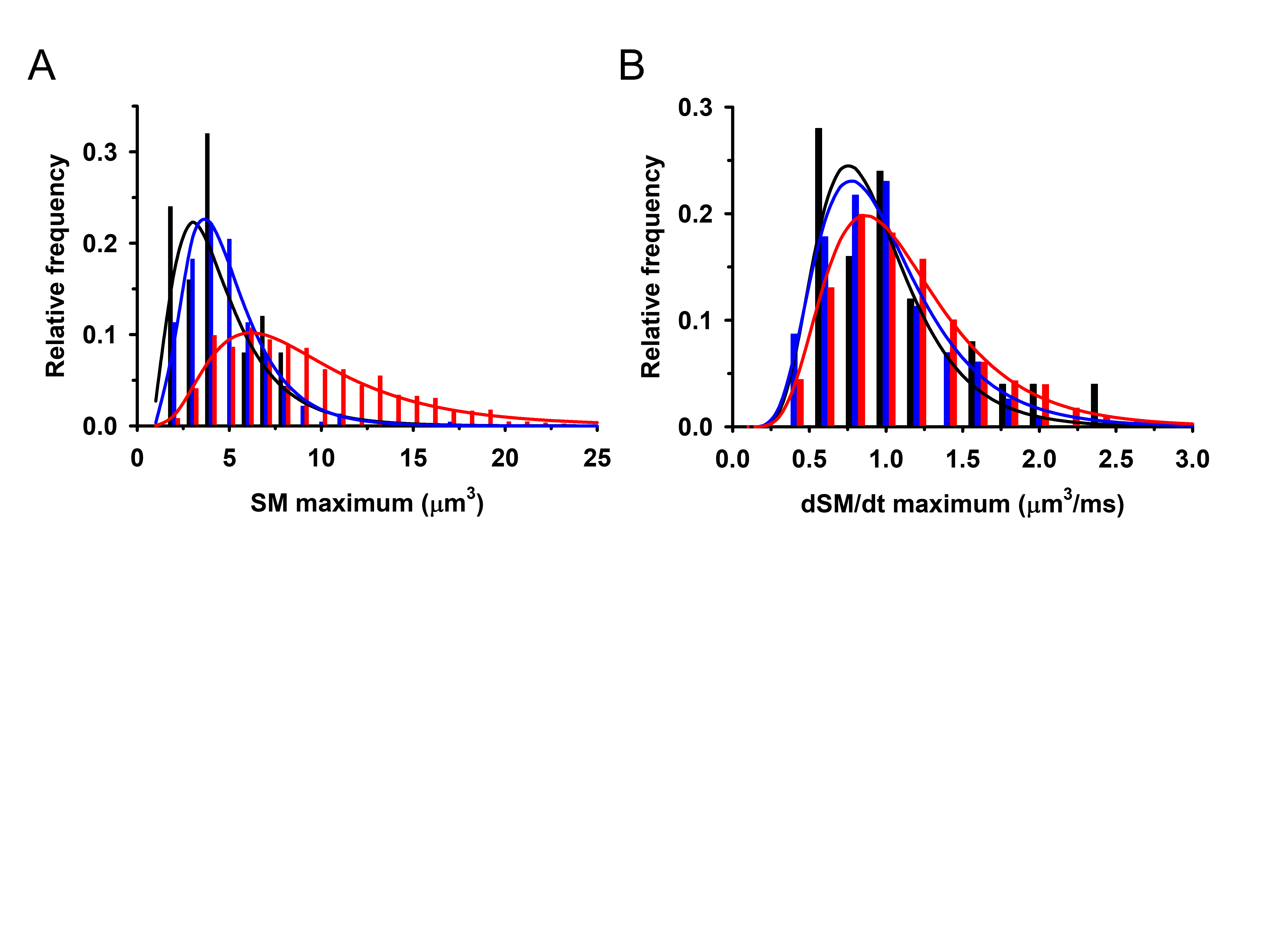

Supplement: Supplementary file 1 [file biomolecules-16-00910-s001.zip › Figure_S2.tif]

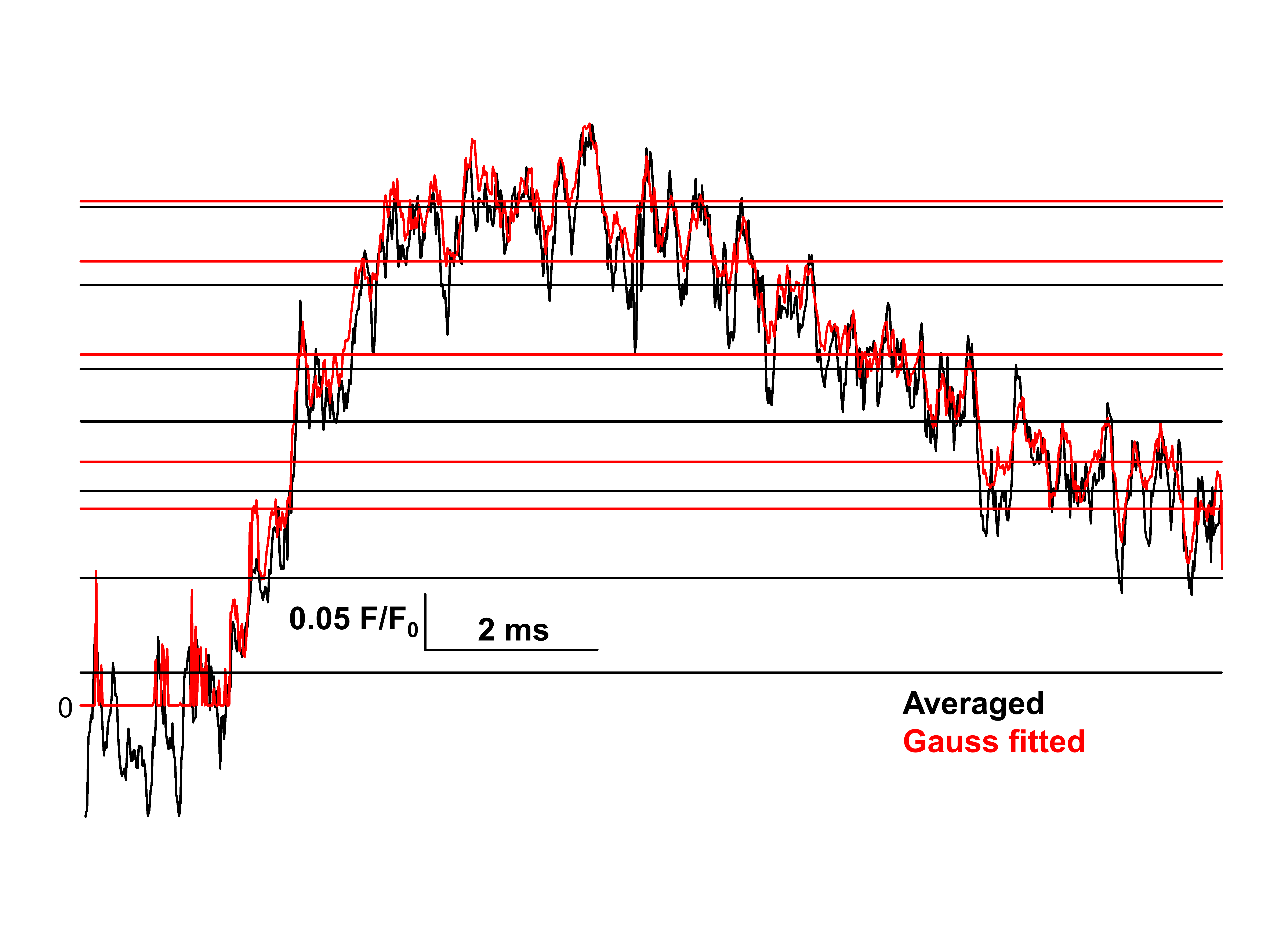

Supplement: Supplementary file 1 [file biomolecules-16-00910-s001.zip › Figure_S3.tif]

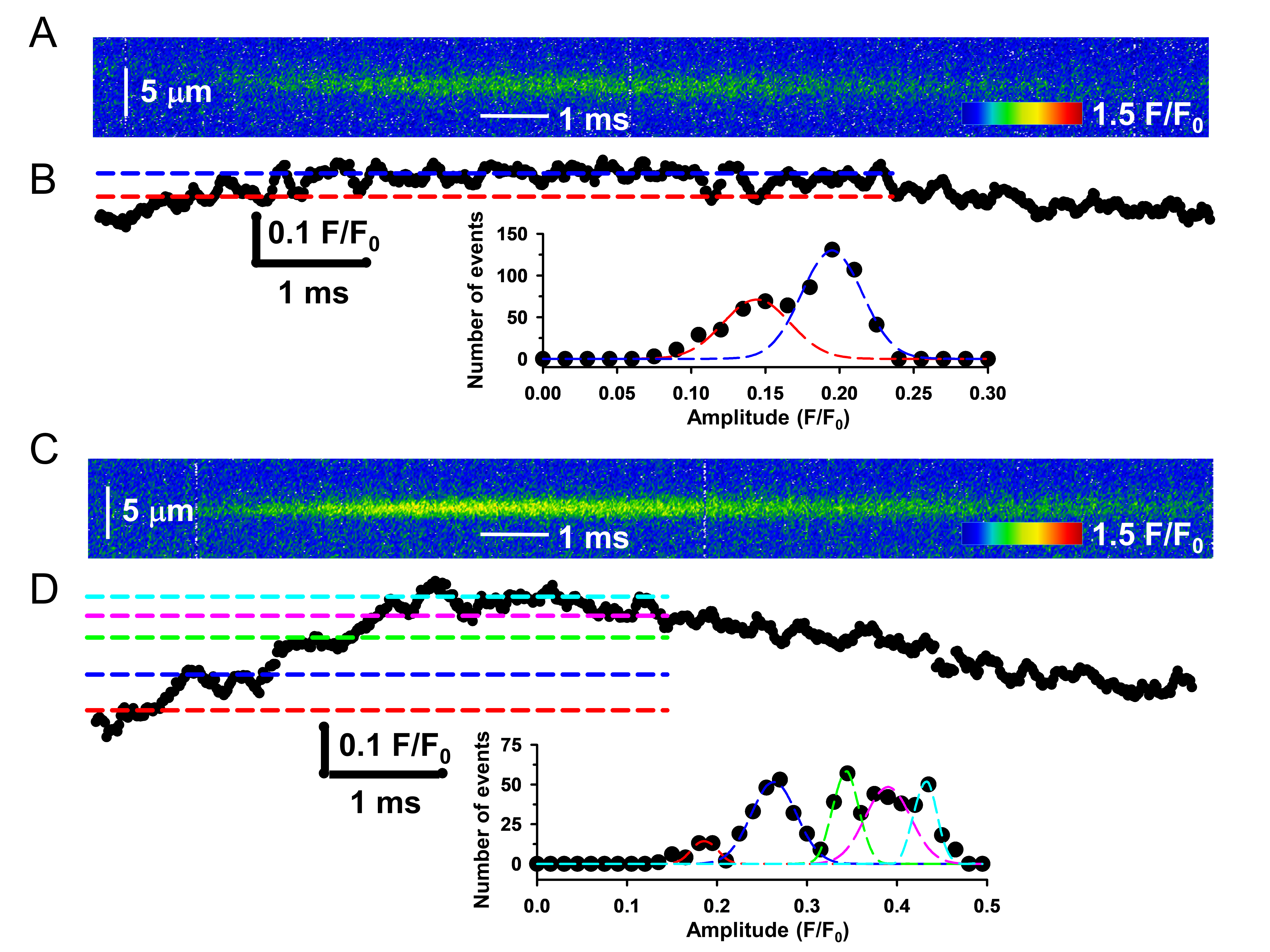

Supplement: Supplementary file 1 [file biomolecules-16-00910-s001.zip › Figure_S4.tif]

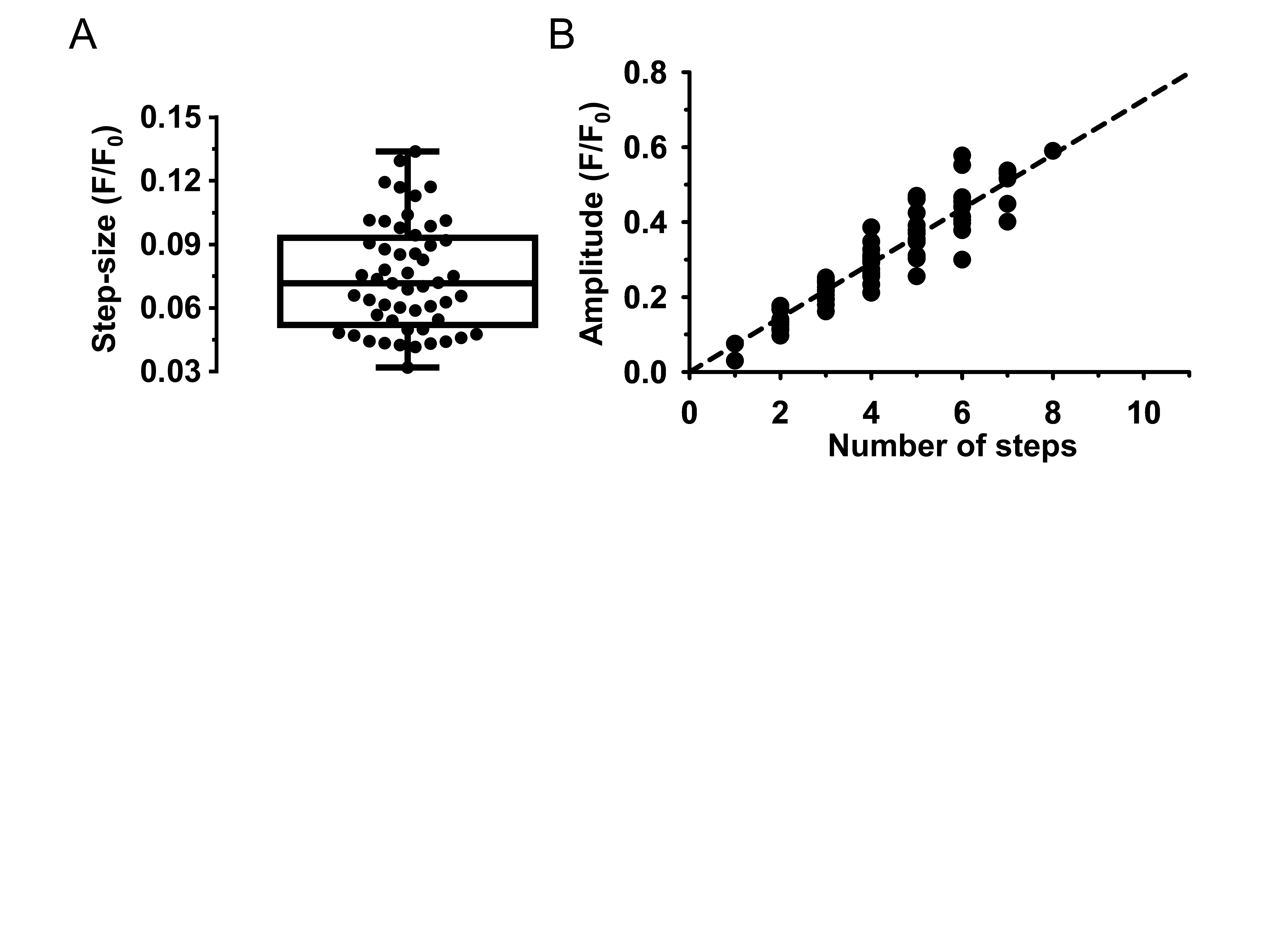

Supplement: Supplementary file 1 [file biomolecules-16-00910-s001.zip › Figure_S5.tif]

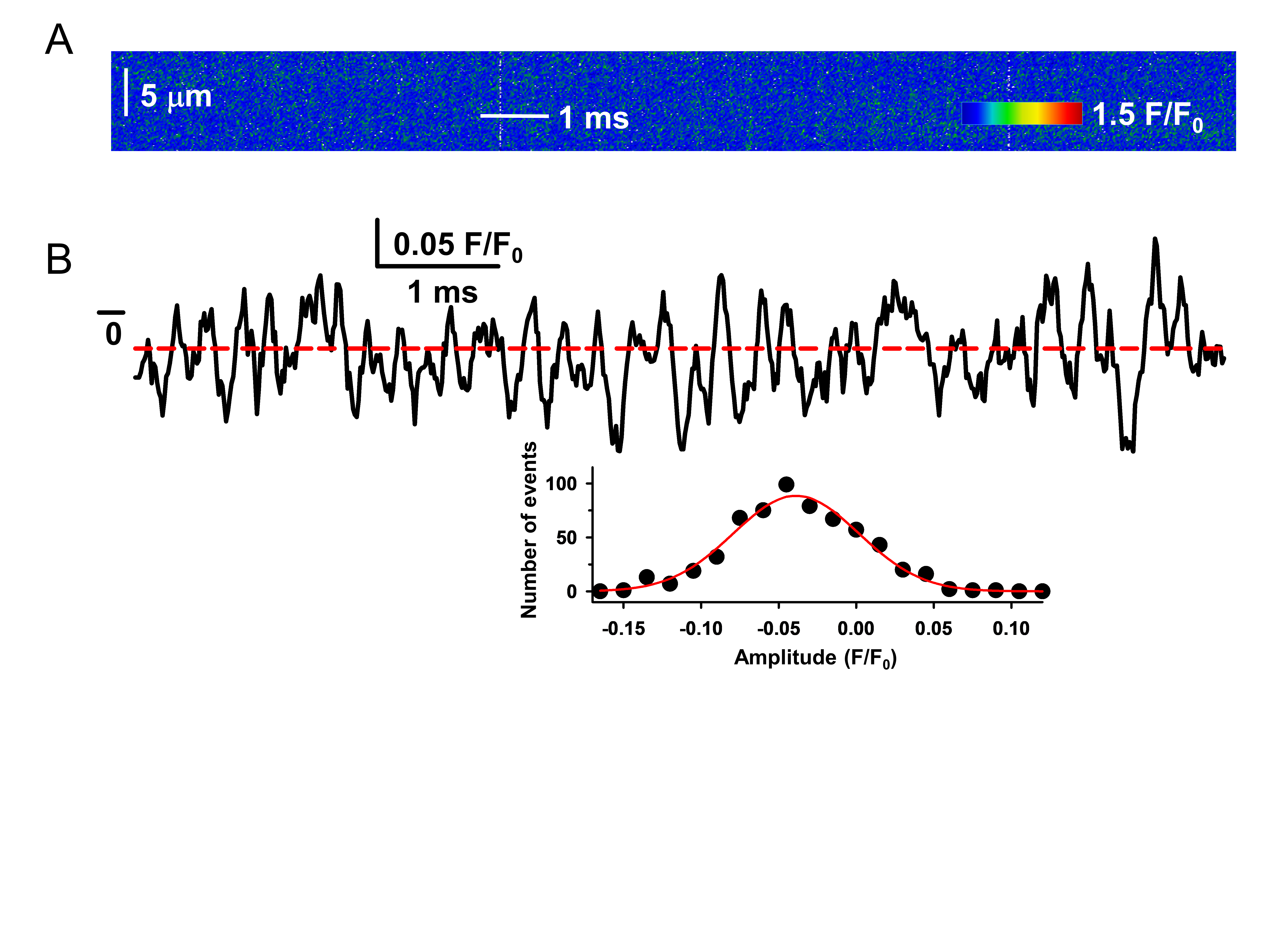

Supplement: Supplementary file 1 [file biomolecules-16-00910-s001.zip › Figure_S6.tif]

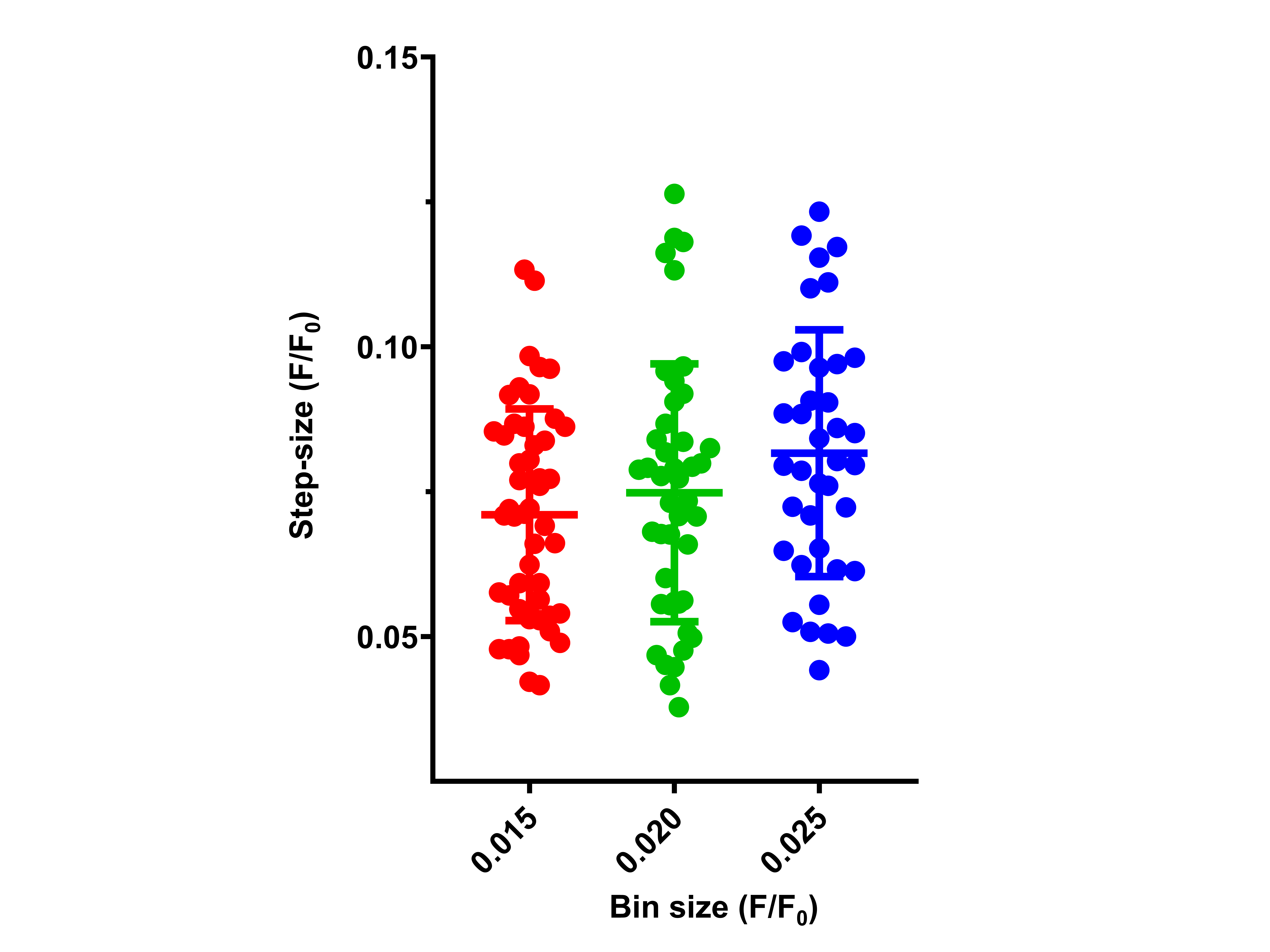

Supplement: Supplementary file 1 [file biomolecules-16-00910-s001.zip › Figure_S7.tif]
